# APP Regulates NGF Receptor Trafficking and NGF-Mediated Neuronal Differentiation and Survival

**DOI:** 10.1371/journal.pone.0080571

**Published:** 2013-11-01

**Authors:** Yun-wu Zhang, Yaomin Chen, Yun Liu, Yingjun Zhao, Francesca-Fang Liao, Huaxi Xu

**Affiliations:** 1 Fujian Provincial Key Laboratory of Neurodegenerative Disease and Aging Research and Institute of Neuroscience, College of Medicine, Xiamen University, Xiamen, China; 2 Neurodegenerative Disease Research Program, Sanford-Burnham Medical Research Institute, La Jolla, California, United States of America; 3 Department of Pharmacology, University of Tennessee Health Science Center, College of Medicine, Memphis, Tennessee, United States of America; University of Florida, United States of America

## Abstract

β-amyloid precursor protein (APP) is a key factor in Alzheimer's disease (AD) but its physiological function is largely undetermined. APP has been found to regulate retrograde transport of nerve growth factor (NGF), which plays a crucial role in mediating neuronal survival and differentiation. Herein, we reveal the mechanism underlying APP-mediated NGF trafficking, by demonstrating a direct interaction between APP and the two NGF receptors, TrkA and p75NTR. Downregulation of APP leads to reduced cell surface levels of TrkA/p75NTR and increased endocytosis of TrkA/p75NTR and NGF. In addition, APP-deficient cells manifest defects in neurite outgrowth and are more susceptible to Aβ-induced neuronal death at physiological levels of NGF. However, APP-deficient cells show better responses to NGF-stimulated differentiation and survival than control cells. This may be attributed to increased receptor endocytosis and enhanced activation of Akt and MAPK upon NGF stimulation in APP-deficient cells. Together, our results suggest that APP mediates endocytosis of NGF receptors through direct interaction, thereby regulating endocytosis of NGF and NGF-induced downstream signaling pathways for neuronal survival and differentiation.

## Introduction

An important pathological hallmark of Alzheimer's disease (AD) is the formation of extracellular senile plaques in the brain, whose major components are β-amyloid (Aβ) peptides. Aβ is proteolytically derived from the β-amyloid precursor protein (APP) through sequential cleavages first by β-secretase (BACE1) and then by the γ-secretase complex [1], [2], [3]. Extensive evidence demonstrates that overproduction/accumulation of Aβ in vulnerable brain regions is a primary culprit in AD pathogenesis: Aβ is neurotoxic and can trigger a cascade of neurodegenerative steps including synaptic dysfunction/loss, formation of intra-neuronal fibrillary tangles, and subsequent neuronal death [4], [5].

Full-length APP is a type-I transmembrane protein. After its synthesis in the endoplasmic reticulum, APP is transported along the secretory pathway to the Golgi/trans-Golgi network and the plasma membrane [6], [7], [8]. Cell surface APP can be internalized for endosomal/lysosomal degradation [9], [10]. Although APP has been under great scrutiny since its identification, the physiological functions of APP remain largely undetermined. A role for APP has been suggested in signal transduction, cell adhesion, calcium metabolism, neurite outgrowth and synaptogenesis, etc, all requiring corroboration with *in vivo* evidence [2]. In addition, several studies, including ours, have indicated that APP may play a role in protein trafficking regulation: APP was found to function as a kinesin-I membrane receptor to mediate axonal transport of BACE1 and PS1 [11], [12], though another study failed to verify this result [13]. We recently found that APP regulates cell surface delivery of γ-secretase components but not BACE1 [14]. APP was also shown to interact with high-affinity choline transporter and APP deficiency affected its endocytosis [15]. Another interesting study found that increased doses of APP markedly decrease retrograde transport of nerve growth factor (NGF) and causes degeneration of forebrain cholinergic neurons in a mouse model of Down's Syndrome (DS) [16].

NGF belongs to the neurotrophin family, which plays an important role in regulating development of both the central and peripheral nervous systems [17]. Neurotrophins bind to specific receptor tyrosine kinases (Trks) at the cell surface and activate them. Formation of the ligand-receptor complexes also initiates internalization of the activated receptors into vesicles and these internalized receptors remain activated as long as they are associated with the ligands [18]. Upon binding to its specific receptors, TrkA and p75NTR, NGF can activate a series of downstream signaling events mediating neuronal survival, differentiation, and maintenance. The two major NGF-mediated signaling pathways, PI3K/Akt and MAPK, are involved in neuronal survival and differentiation, respectively [19], [20], [21]. Since retrograde transport of NGF after endocytosis upon its binding to TrkA/p75NTR was shown to be affected by APP and the underlying mechanism has not been determined [16], herein we investigate the effects of APP on regulating TrkA/p75NTR trafficking and on the downstream signaling events upon NGF stimulation.

## Materials and Methods

### Cell cultures, transfection and infection

Maintenance of mouse embryonic fibroblast (MEF) cells derived from *APP/APLP2* double knockout and control mice [22], phenochromocytoma PC12 cells [17], and primary neuronal cultures derived from postnatal day 0 mice or embryonic day 17 rat embryos [23], has been previously described. MEF cells were transiently transfected with APP, TrkA, and/or p75NTR plasmids using Lipofectamine 2000 (Invitrogen). Stable downregulation of APP in PC12 cells was achieved by transfection of a pSUPER RNAi vector containing a small hairpin RNA (shRNA) targeting the APP sequence and selection with 200 µg/mL G418 [14]. Lentivirus containing the same APP targeting shRNA sequence was used to infect primary rat neurons for APP downregulation. All procedures were performed in accordance with the Guide for Care and Use of Laboratory Animals of the National Institutes of Health and were approved by the Institutional Animal Use and Care Committee of Sanford-Burnham Medical Research Institute.

### Antibodies

Antibodies used in this study included 22C11 recognizing the amino-terminus of APP (Chemicon), 369 recognizing the carboxyl-terminus of APP, different TrkA antibodies (Santa Cruz, Chemicon, and Upstate), and p75NTR antibodies (Abcam and Cell Signaling). Antibodies recognizing Akt, phosphorylated Akt, MAPK, phosphorylated MAPK, and MAP2 were from Cell Signaling.

### Cell surface protein biotinylation

Biotinylation was carried out as previously described [14]. Biotin-labeled cell surface proteins were precipitated with streptavidin-agarose beads (Pierce), subjected to SDS-PAGE, and analyzed by Western blotting with indicated antibodies.

### Co-immunoprecipitation

PC12 cells were lysed with CelLytic M Cell Lysis Reagent (Sigma) along with a protease inhibitor cocktail (Roche). Cell lysates were subjected to immunoprecipitation with the indicated antibodies and rProtein A-sepharose beads (Biochain Institute), followed by Western blotting.

### NGF treatments

To study the endocytosis of NGF, PC12 cells with stable downregulation of APP and control cells were treated with 1 nM quantum dot-labeled NGF (QD-NGF) for 3 h [24]. After a complete wash, cells were fixed, permeabilized, stained with DAPI, and observed under a fluorescent microscope. In addition, cells were treated with 100 ng/mL NGF for different time periods and the levels of phosphorylated and total Akt/MAPK were analyzed by Western blotting.

### NGF receptor endocytosis

To study the endocytosis of p75NTR and TrkA, cells were first incubated with primary antibodies against p75NTR or TrkA at 4°C for 1 h, and then treated with 100 ng/mL NGF at 37°C for 1 h. Cells were then fixed and incubated with a secondary antibody conjugated with Alexa Fluor®-594 (for detecting cell surface proteins) for 1 h. After a complete wash, cells were permeabilized and then incubated with another secondary antibody conjugated with Alexa Fluor-488 (for detecting both cell surface and internalized proteins). Finally, cells were observed under a confocal microscrope.

### Neurite outgrowth

The next day after plating of embryonic day 17 rat primary neurons, neurons were infected with APP or scrambled control (SC) RNAi-containing lentivirus for 1 d. These neurons were then treated with or without 100 ng/mL NGF for 5 d, and then fixed, permeabilized, immunostained with MAP2 antibody and fluorescence-labeled secondary antibody, and observed under a fluorescent microscope. The neurite lengths of infected (indicated by GFP fluorescence) neurons (indicated by positive MAP2 staining) were measured for comparison.

### Neuronal death

Neurons derived from postnatal day 0 APP heterozygous mice and rat primary neurons with APP downregulated by RNAi, as well as respective controls, were treated with or without 100 ng/mL NGF for 5 d. These neurons were then treated with 25 µM Aβ for 1 d. Samples were stained by propidium iodide. The numbers of dead (indicated by positive PI staining) neurons were counted and compared.

## Results

### APP interacts with TrkA and p75NTR and regulates their cell surface accumulation

**Figure 1:**
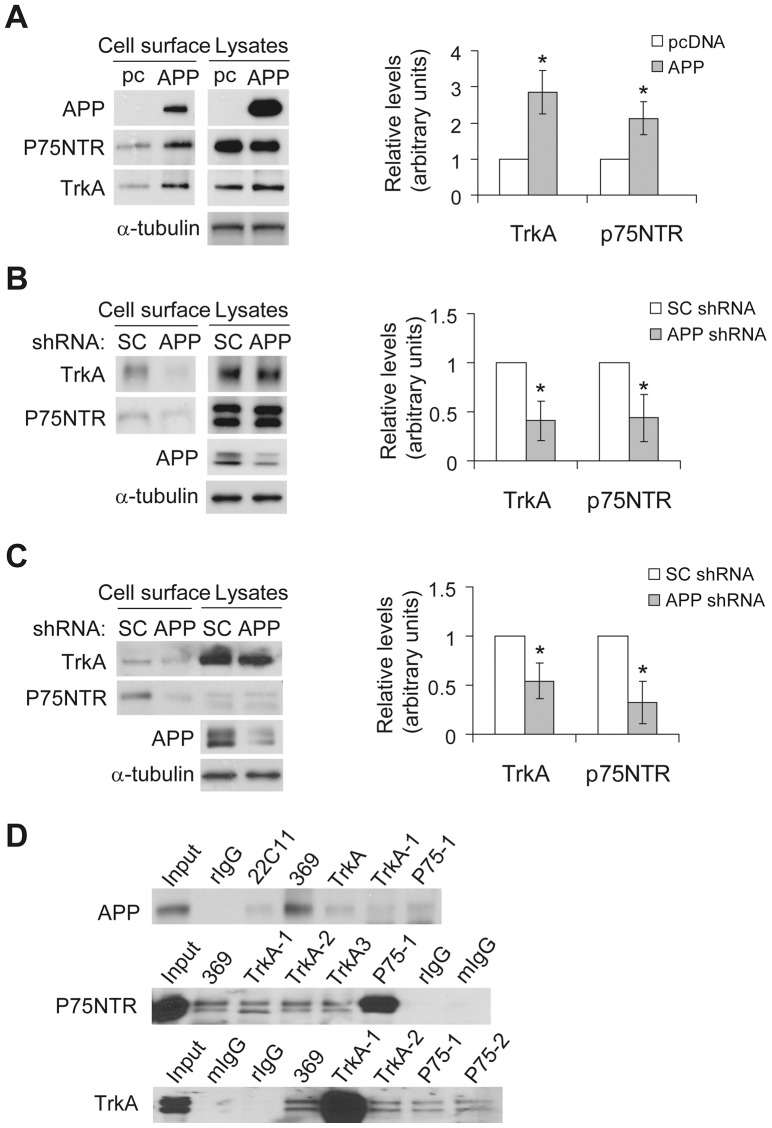
APP regulates cell surface levels of NGF receptors. (A) APP/APLP2 dKO cells were first transfected with p75NTR or TrkA. After splitting equally, cells were transfected with pc DNA (control) or APP. Cell surface proteins were biotinylated, affinity precipitated, and subjected to Western blotting. (B) PC12 cells were stably transfected with APP or scrambled control (SC) shRNA-containing vectors. Cell surface protein levels were analyzed by biotinylation. (C) Rat primary neurons were infected with APP or scrambled control (SC) shRNA-containing lentivirus for 5 d. Cell surface protein levels were analyzed by biotinylation. Protein levels were quantified by densitometry and normalized to those of controls for comparison (set as one arbitrary unit). Error bars indicate SEM. *: *P*<0.05, n = 3. (D) Equal protein amounts of PC12 cell lysates were incubated with rabbit IgG (rIgG), mIgG, APP antibodies (22C11 and 369), different TrkA antibodies (1–3) and P75NTR antibodies (1–2). Immunoprecipitated proteins were subjected to Western blotting.

While the underlying mechanism remains undetermined, it has been shown that APP overexpression impairs the retrograde axonal transport of NGF [16]. Because endocytosis of NGF is the first step for its retrograde transport and NGF endocytosis is mediated by its binding to the NGF receptors, TrkA and p75NTR, at the cell surface, we investigated whether APP can regulate cell surface levels of TrkA and p75NTR. We first overexpressed TrkA and p75NTR individually in APP/APLP2 double knockout MEF cells and then transfected them with APP or control pcDNA. The results showed that the steady state cell surface levels of TrkA and P75NTR were increased by 2.9 and 2.1 folds, respectively, in the presence of APP (Figure 1A). We also generated stable cell lines of rat phenochromocytoma PC12 cells in which the level of APP was downregulated by RNAi and found that these cells had reduced steady state cell surface levels of TrkA (∼2.4 folds) and P75NTR (∼2.3 folds) (Figure 1B). In addition, downregulation of APP in rat primary neurons by RNAi also drastically reduced steady state cell surface levels of TrkA (∼1.8 folds) and p75NTR (∼3.1 folds) (Figure 1C). Together these results clearly indicate that APP can regulate cell surface levels of the NGF receptors TrkA and p75NTR.

APP has been reported to be able to interact with p75NTR and TrkA [25], [26]. Herein, we carried out co-immunoprecipitation studies and confirmed that APP indeed interacts with p75NTR and TrkA (Figure 1D). Fluorescent immunostaining also showed that APP colocalizes with TrkA and p75NTR (data not shown).

### Downregulation of APP results in increased endocytosis of NGF and NGF receptors

**Figure 2:**
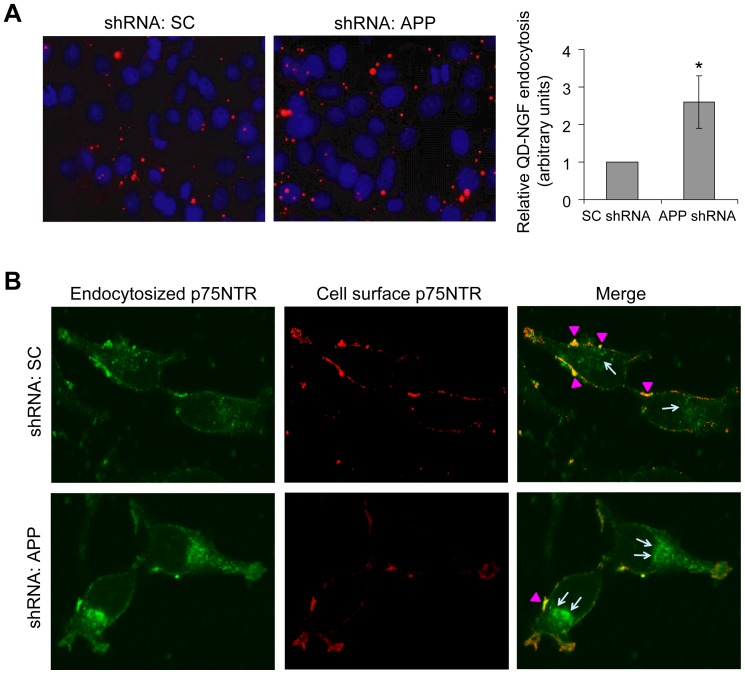
APP deficiency promotes endocytosis of NGF and NGF receptors. (A) PC12 cells stably expressing APP shRNA and control cells expressing scrambled control (SC) shRNA were treated with 1 nM QD-NGF for 3 h. After a complete wash, cells were fixed, permeabilized, stained with DAPI, and observed under a fluorescent microscope. Residual QD-NGF (in red) levels were quantified from 5 randomly selected regions with comparable cell numbers. Control was set as one arbitrary unit. *: *P*<0.05. (B) Cells were first incubated with a p75NTR antibody at 4°C for 1 h, and then treated with 100 ng/mL NGF at 37°C for 1 h. Cells were fixed and incubated with a secondary antibody conjugated with Alexa Fluor®-594 for 1 h. After a complete wash, cells were permeabilized and then incubated with another secondary antibody conjugated with Alexa Fluor-488. Cells were observed under a confocal microscrope. Arrows and arrow heads indicate internalized and cell surface p75NTR, respectively.

Next, we studied whether APP deficiency affects NGF endocytosis. When PC12 cells with APP stably downregulated by RNAi were treated with QD-NGF for 3 h, the level of endocytosed QD-NGF in these cells was about 2.6 folds higher than that in control cells (Figure 2A). These results are consistent with the finding that APP overexpression impairs retrograde transport of NGF [16].

Binding of NGF to its receptors is necessary for its endocytosis. Therefore, we studied whether APP also affects endocytosis of NGF receptors. After cells were treated with NGF for 1 h, we observed considerably more internalized p75NTR in APP downregulated cells than in control cells (Figure 2B). A similar finding was observed for TrkA endocytosis (data not shown). These results suggest that APP may mediate NGF endocytosis through regulating endocytosis of TrkA/p75NTR.

### APP deficiency results in increased neuronal differentiation and survival in response to NGF

**Figure 3:**
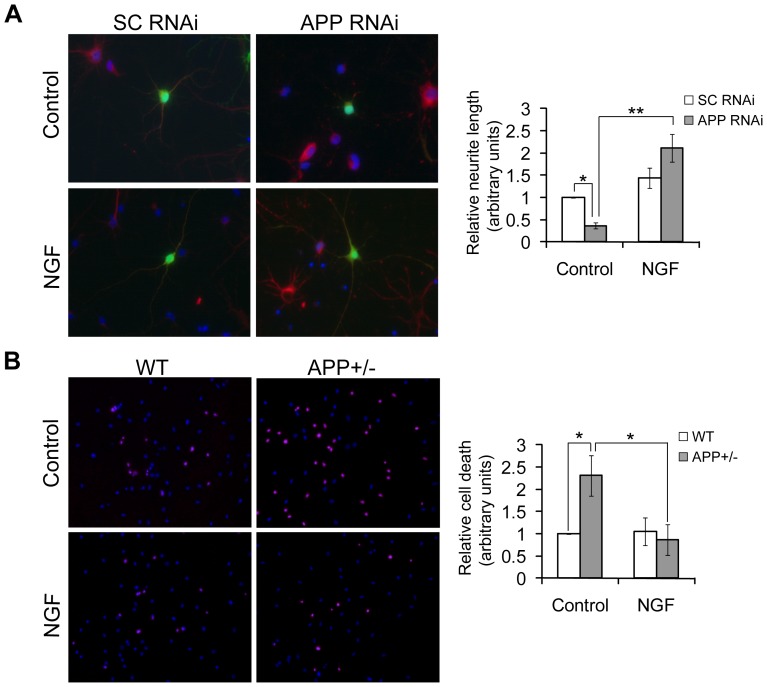
APP deficiency impairs neurite outgrowth and neuronal survival at basal levels, but promotes neurite outgrowth and neuronal survival more acutely upon NGF stimulation. (A) The day after plating embryonic day 17 rat primary neurons, neurons were infected with APP or scrambled control (SC) RNAi-containing lentivirus for 1 d. These neurons were then treated with or without 100 ng/mL NGF for 5 d, fixed, permeablized, immunostained with MAP2 antibody and a fluorescent-labeled secondary antibody, and observed under a fluorescent microscope. Infected cells were indicated by GFP fluorescence (in green) and neurons were indicated by positive MAP2 stainining (in red). The neurite lengths of infected neurons (>100) were measured for comparison. (B) Primary neurons from postnatal day 0 wild type (WT) and APP heterozygous (+/−) mice were treated with or without 100 ng/mL NGF for 5 d. These neurons were then treated with 25 µM Aβ for 1 d. After staining with propidium iodide (in red) and DAPI (in blue), the numbers of dead neurons (>300) were counted for comparison. Controls were set as one arbitrary unit. Error bars indicate SEM. *: *P*<0.05, **: *P*<0.01.

Since NGF activates a series of downstream signaling events that mediate neuronal survival and differentiation, we investigated whether altering cellular levels of APP affects neuronal survival and differentiation. In the absence of exogenous NGF treatment (i.e. at the basal levels), neurite outgrowth (indicative of neuronal differentiation) of rat primary neurons with APP downregulated by RNAi was about 2.7 folds less than that of control cells (Figure 3A), consistent with our previous results that APP-deficient mouse neurons manifest dramatic neuronal outgrowth defects [27]. However, when neurons were treated with NGF for 5 d, neurite outgrowth of APP-downregulated neurons was about 1.5 folds more than that of control cells (Figure 3A), suggesting that APP deficiency results in an increased neuronal differentiation in response to NGF treatment. We also compared neurons' resistance to Aβ neurotoxicity. The results showed that when cells were treated with Aβ, APP heterozygous knockout (+/−) mouse neurons had a much higher death rate (∼2.3 folds) than control neurons in the absence of NGF treatments (i.e., at the basal NGF levels) (Figure 3B), but APP+/− neurons had a similar death rate to control neurons upon NGF treatments. These data suggest that APP-deficient cells respond more acutely to NGF-mediated survival signals than control cells.

### Downregulation of APP enhances the NGF-mediated PI3K/Akt and MAPK pathways

**Figure 4:**
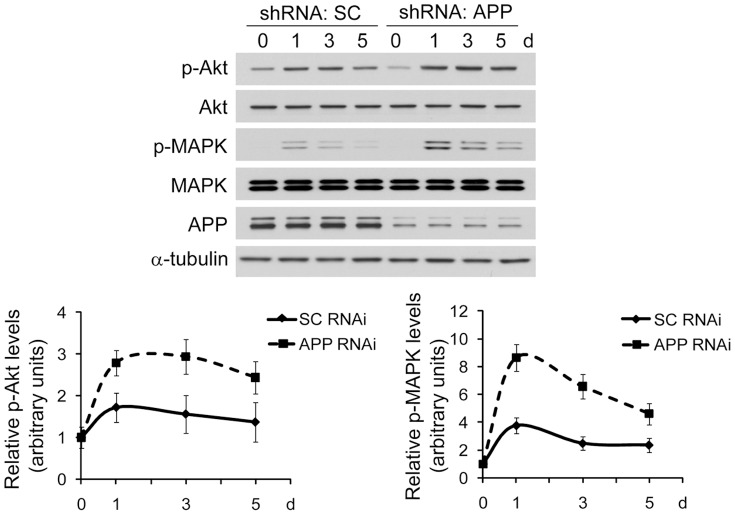
APP deficiency enhances activation of Akt and MAPK upon NGF treatment. PC12 cells stably expressing APP shRNA and control cells expressing scrambled control (SC) shRNA were treated with 100 ng/mL NGF for the indicated time periods. Equal protein amounts of cell lysates were subjected to Western blotting to measure phosphorylation (p)/activation of Akt and MAPK. Protein levels were quantified by densitometry and normalized to those of controls for comparison (set as one arbitrary unit). Error bars indicate SEM, n = 3.

Neuronal survival and differentiation are regulated by the NGF-activated downstream signaling pathways PI3k/Akt and MAPK, respectively [19], [20], [21]. Herein, we found that when cells were treated with NGF, phosphorylation of both Akt and MAPK for their activation was dramatically elevated (Figure 4): in control cells, NGF treatments for 1, 3 and 5 d promoted Akt phosphorylation for 1.7, 1.6 and 1.4 folds, respectively, and promoted MAPK phosphorylation for 3.8, 2.5 and 2.4 folds, respectively; while in APP downregulated cells, NGF treatments for 1, 3 and 5 d promoted Akt phosphorylation for 2.8, 2.9 and 2.4 folds, respectively, and promoted MAPK phosphorylation for 8.7, 6.6 and 4.6 folds, respectively. However, when we compared the change of Akt and MAPK phosphorylation in control and in APP downregulated cells, we noticed that the increased levels of both Akt and MAPK phosphorylation were much higher in APP downregulated cells than in control cells (Figure 4), which is consistent with the more significant survival and differentiation responses to NGF in these cells.

## Discussion

Although its detailed physiological/pathological function remains largely undetermined, APP is crucially involved in AD as the precursor of Aβ. In addition, a reduced availability of NGF has also been found to contribute to AD: an impairment of NGF maturation from its precursor proNGF causes the vulnerability of cholinergic neurons in AD [28], [29], [30]; deprivation of NGF leads to AD-like pathologies such as Aβ accumulation/deposition, tau hyperphosphorylation, synaptic dysfunction and memory deficits in mice [31], [32]; and administration of NGF can ameliorate Aβ pathologies and prevent memory deficits in AD animal models [33], [34]. Recent studies have suggested a correlation between APP processing/Aβ accumulation and NGF/NGF receptor mediated signaling pathways. For example, our present study, as well as others' has shown that APP can interact with both TrkA and p75NTR [25], [26]. One study suggested that the interaction between APP and TrkA requires the tyrosine residue at APP position 682 (Y682, numbering based on APP695 isoform) [26]. APP-Y682 has been shown to be important for the function and processing of APP [35]. Interestingly, overexpression of TrkA has been found to be associated with both phosphorylation of APP-Y682 and alteration of APP processing [36]. In addition, there are reports showing that NGF can affect APP expression and localization [37], [38], [39]. On the other hand, APP can regulate activation of the NGF/TrkA signaling pathway, the subcellular distribution of TrkA and the sensitivity of neurons to the trophic action of NGF [26]. Increased levels of APP also markedly decreases retrograde transport of NGF and causes degeneration of forebrain cholinergic neurons in a mouse model of DS [16]. However, the detailed molecular pathways linking APP and NGF/NGF receptor signaling have yet to be fully clarified.

Herein, we have found that APP deficiency results in a significant decrease in cell surface levels of the two NGF receptors, TrkA and p75NTR. Because APP has been shown to mediate intracellular trafficking of certain proteins [11], [12], [14], [15], one possibility is that APP can also regulate intracellular trafficking of TrkA and p75NTR through its interaction with these receptors. Therefore, an increase in the APP level could result in more TrkA/p75NTR at the cell surface and thus inhibit NGF endocytosis, whereas a decrease in the APP level could facilitate endocytosis of NGF upon its binding to TrkA and p75NTR. Indeed, our data have shown that endocytosis of TrkA/p75NTR, as well as endocytosis of NGF, is drastically higher in APP-downregulated cells than in control cells.

Upon binding NGF, cell surface receptors are activated and trigger a series of downstream signaling pathways, such as PI3K/Akt and MAPK, which mediate neuronal survival and differentiation, respectively [19], [20], [21]. Herein, we have found that upon NGF treatment, Akt and MAPK phosphorylation/activation is much higher in APP-downregulated cells than in control cells. This is probably attributed to an increased endocytosis of NGF-receptor complexes in APP-downregulated cells and these complexes remain active as long as the ligand keeps associated with the receptors [18]. Moreover, more extensive activation of Akt and MAPK signaling pathways in APP-deficient neurons facilitates their differentiation and survival in response to NGF: although APP-deficient neurons have significant defects in neurite outgrowth and are highly susceptible to neurotoxicity-induced neuronal death when compared to control cells, as shown in our results (Figure 3) and described previously [27], these neurons have comparable neurite outgrowth and Aβ-induced death rates to those of control cells.

Together, our results show that APP interacts with TrkA/p75NTR, thereby regulating cell surface levels of TrkA/p75NTR and their endocytosis, as well as endocytosis of NGF, and affecting the NGF-mediated signaling cascades for neuronal survival and differentiation. Consistently, APP has been implicated in critical neuronal functions such as synapse formation, growth cone outgrowth and axon guidance [2], [3]. Hence dysregulated NGF signaling cascades following APP impairment may lead to the pathogenic states, including AD and DS.
